# Supplementary figures and images for: The impact of a competitive event and the efficacy of a lactic acid bacteria-fermented soymilk extract on the gut microbiota and urinary metabolites of endurance athletes: An open-label pilot study
Source: PLoS One. 2022 Jan 27;17(1):e0262906. doi: 10.1371/journal.pone.0262906 (PMC8794134; doi:10.1371/journal.pone.0262906)

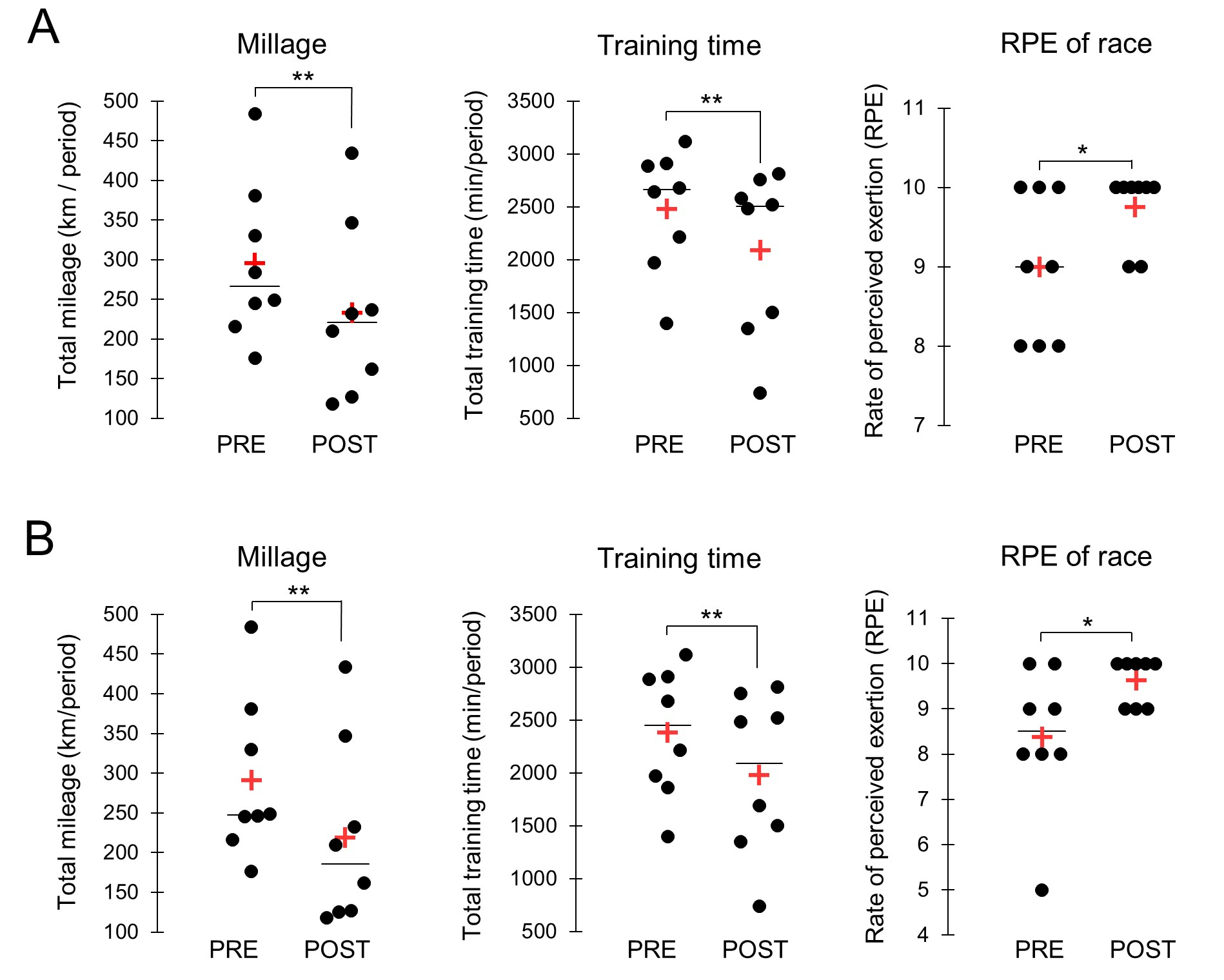

Supplement: S1 Fig — Total mileage, training time, and rate of perceived exertion (RPE) are shown. (A) Exercise load of 8 participants in the analysis of fecal microbiota. n = 8 (5 male /3 female). (B) Exercise load of 8 participants in the analysis of urinary metabolites. n = 8 (6 male/2 female). PRE: pre-observation period. POST: LEX-ingestion period. Values represent scatter plot with median (black line) and mean (Red +). Statistical significance was determined using a paired t-test for millage and training time. Statistical significance was determined using the Wilcoxon signed-rank test for RPE. Significant differences between PRE and POST: *p < 0.05. (JPG) [file pone.0262906.s001.jpg]

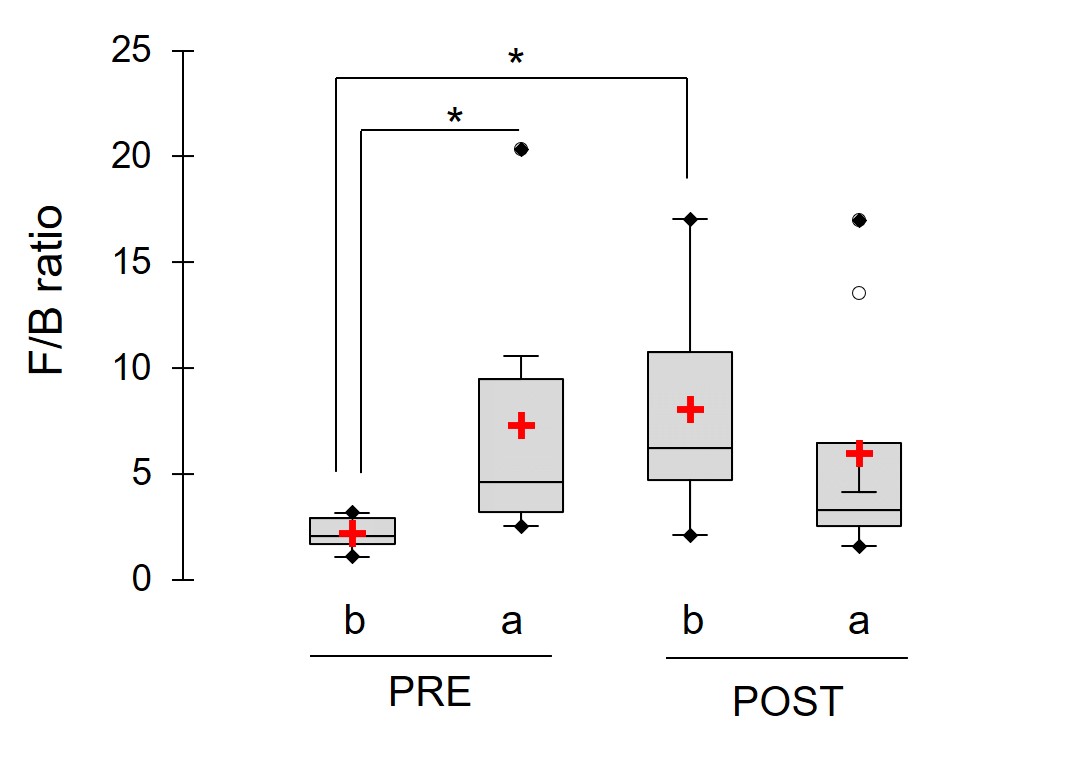

Supplement: S2 Fig — POST: LEX-ingestion period. b: Before the race. a: After the race. Values represent box-and-whisker plots with mean (Red +). Statistical significance was determined using Friedman’s test with a Scheffé’s multiple comparison method. * indicate a significant difference of p < 0.05. (JPG) [file pone.0262906.s002.jpg]

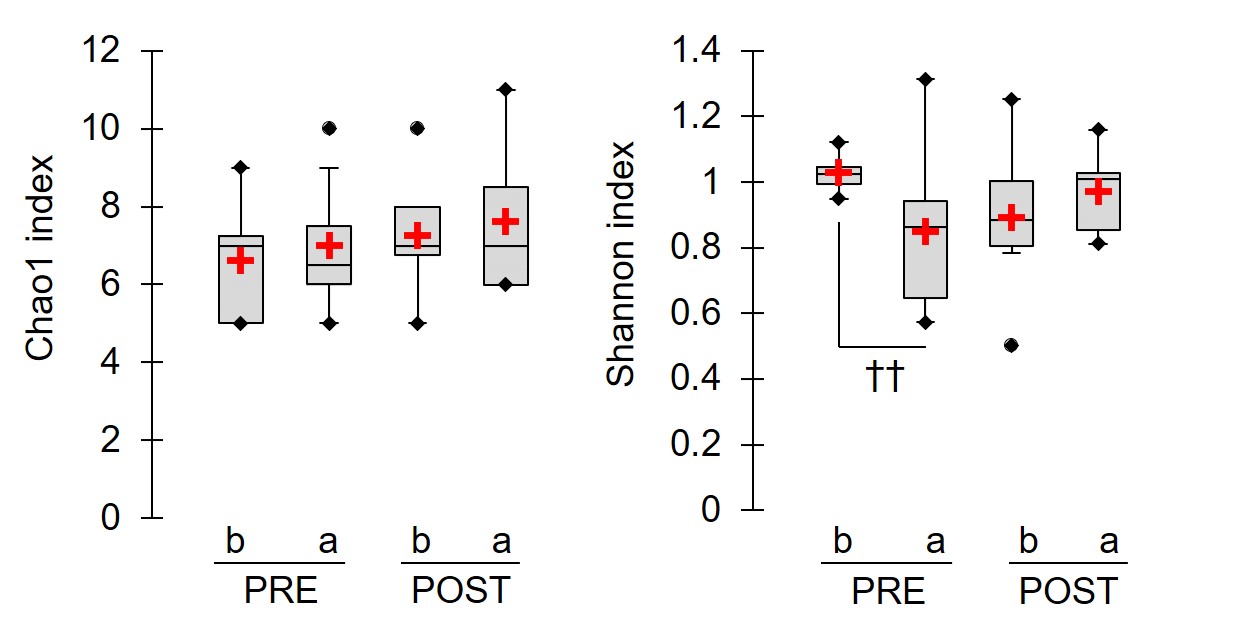

Supplement: S3 Fig — PRE: Pre-observation period. POST: LEX-ingestion period. b: Before the race. a: after the race. Values represent box-and-whisker plots with mean (Red +). Statistical significance was determined using repeated measures ANOVA with Bonferroni’s correction. The difference in variance was determined using Fisher’s F-test. †† indicate a different variance between the two samples: p < 0.01. (JPG) [file pone.0262906.s003.jpg]

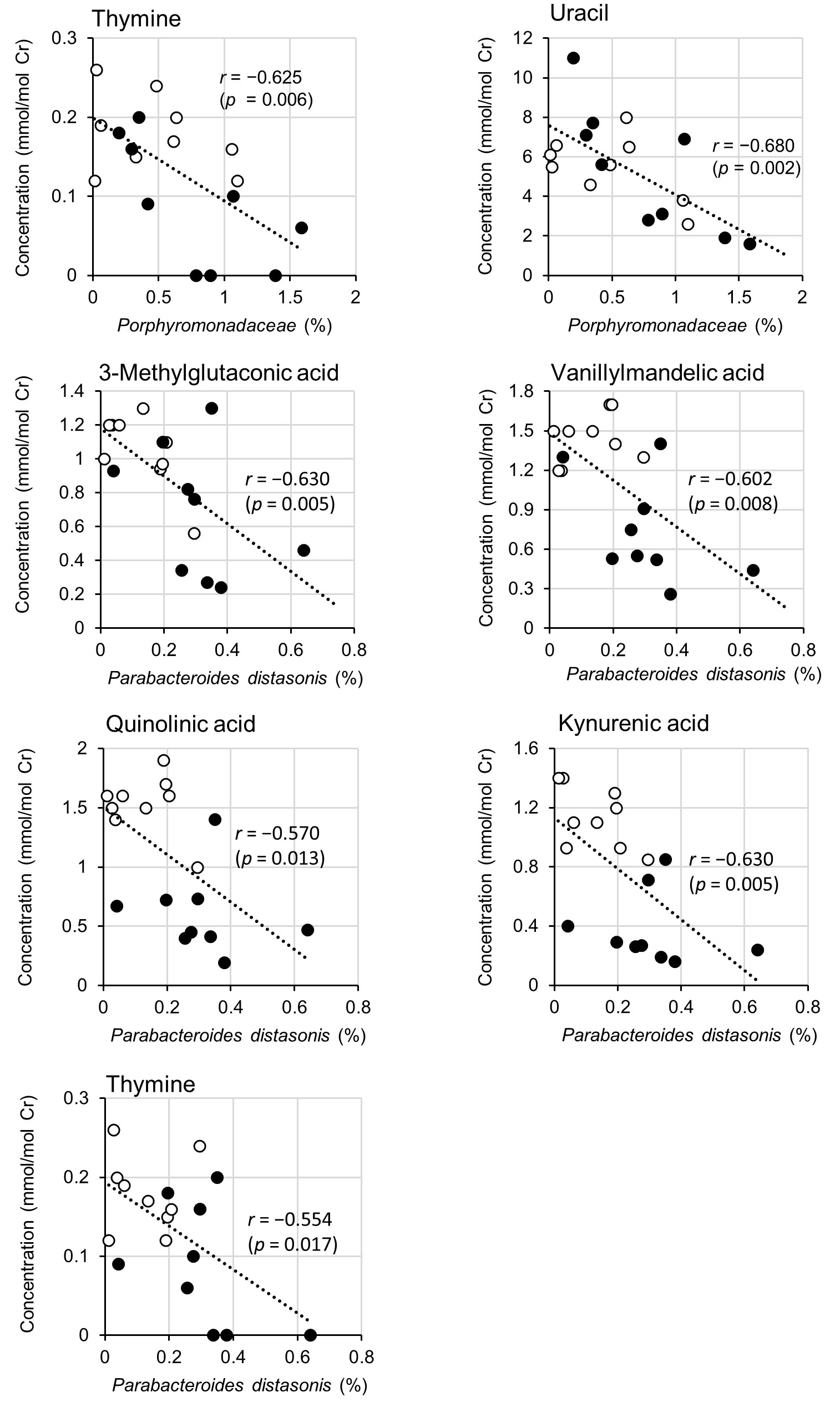

Supplement: S4 Fig — The results of Porphyromonadaceae and Parabacteroides distasonis are shown. Open and closed circles represent values on the pre-observation period (PRE) and LEX-ingestion period (POST), respectively. Pearson’s correlation coefficients (r) are shown for each plot with p-values. The analysis was performed using data from 18 points of nine participants. (JPG) [file pone.0262906.s004.jpg]

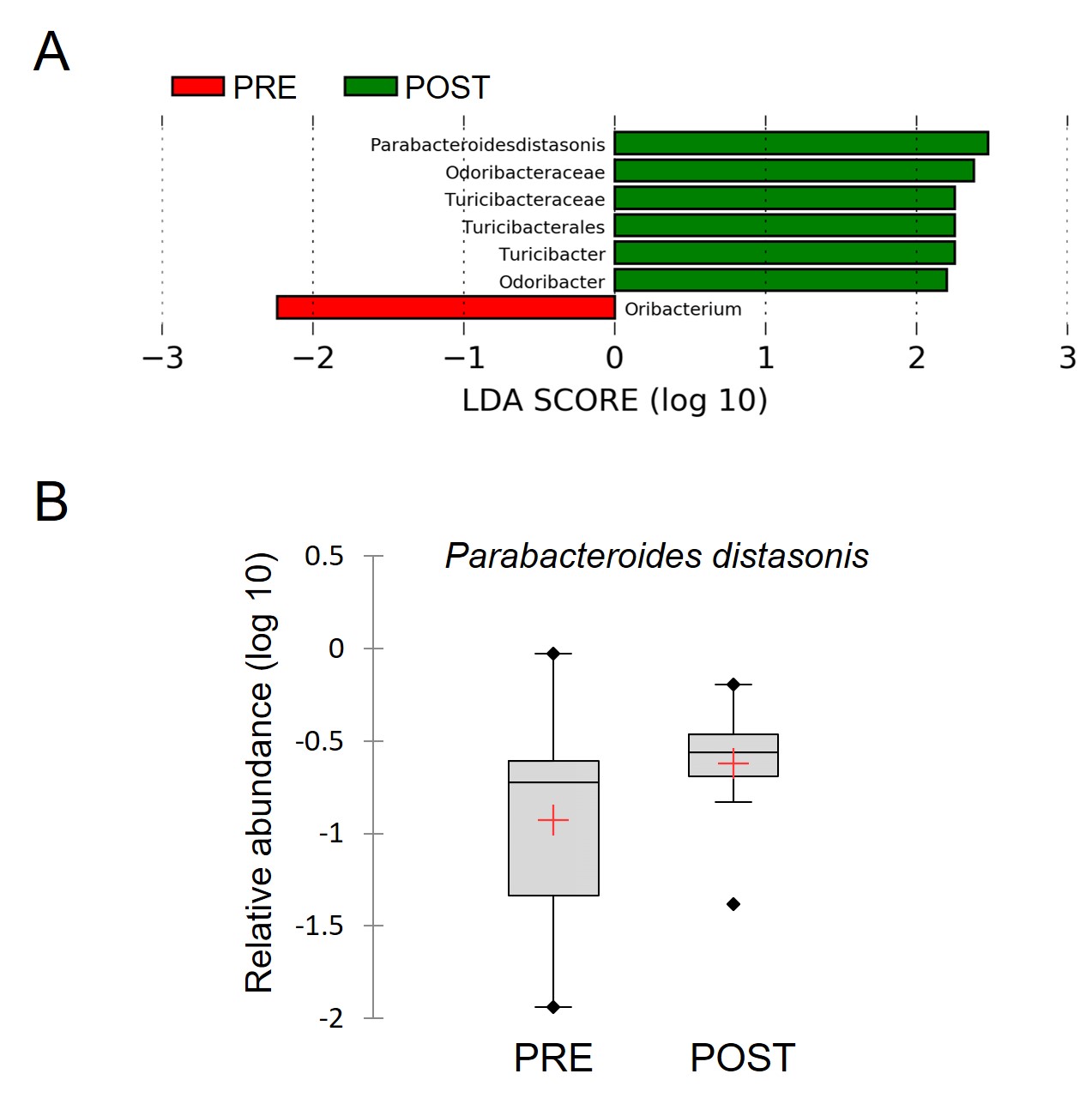

Supplement: S5 Fig — (A) Linear discriminant analysis (LDA) effect size (LEfSe) analysis plot of taxonomic biomarkers in fecal microbiota between PRE_a and POST_a. LDA scores (log10) > 2 and p < 0.05 are listed. (B) Histogram of the relative distribution of Parabacteroides distasonis. Values represent box-and-whisker plots with mean (Red +). The analysis included 11 participants who had data for fecal microbiota before and after LEX ingestion. (JPG) [file pone.0262906.s005.jpg]
