# Supplementary material for: The impact of a competitive event and the efficacy of a lactic acid bacteria-fermented soymilk extract on the gut microbiota and urinary metabolites of endurance athletes: An open-label pilot study
Source: PLoS One. 2022 Jan 27;17(1):e0262906. doi: 10.1371/journal.pone.0262906 (PMC8794134; doi:10.1371/journal.pone.0262906)
Supplement: Attachment — Submitted filename: Response to Reviewers.doc [file pone.0262906.s006.doc]

Krzysztof Durkalec-Michalski, Ph.D

Academic Editor

PLOS ONE

Yasuhiro Sasuga, Ph.D

Hachioji Center for Research & Development

B&S Corporation Co., Ltd.

8^th^ July 2021

Dear Durkalec-Michalski

**Response to reviewers’ comments [PONE-D-21-11683]**

Thank you for reviewing our manuscript and for the helpful comments provided.

Please find enclosed our response to the comments raised. The line numbers provided here are based on our revised manuscript with tracked changes.

1. Reviewer 1

- The authors presented a very interesting pilot study in which they evaluated the effects of lactic acid bacteria-fermented soymilk extract on the microbiome and metabolome of athletes before and after exercise during competition. The researchers made several interesting observations; however, the conclusions they drew are not appropriate. The most important conclusions are that the primary and secondary aims and research hypotheses should be precisely defined; a randomized placebo-controlled study should be conducted (comparator without bacteria); the sample size should be calculated before the study (in the pilot study, authors obtained some data, which will be helpful to do it). Moreover, physiological data on exercise and clinical data on the gastrointestinal tract must be included in the analysis, and it is necessary to record the diet of athletes. The discussion should be rewritten in this direction.

We appreciate the reviewer’s comment. We have divided the conclusions into both primary and secondary. We reflected that in abstracts (line 46–51) and discussion (line 418–425). In the discussion section, we added the limitation of this study and stated that it is necessary to monitor physiological data on exercise and clinical data on the GI tract and/or diet (line426-440). Also, we have noted that a randomized placebo-controlled study should be conducted in the future (line 440–443).

- In addition, several comments on the manuscript should be included:

line 60: Please cite the following position: Kulecka M et al. The composition and richness of the gut microbiota differentiate the top Polish endurance athletes from sedentary controls. Gut Microbes. 2020 Sep 2;11(5):1374-1384.

We accepted this reviewer’s suggestion. Reference 8 (line 73 and line 738–743).

- Line 69: Not only the GI tract is the target of probiotics; please inform readers about other benefits for athletes receiving probiotics.

We accepted this reviewer’s suggestion. We added some key words (stress, host immunity, upper respiratory tract infections). Line 78–80.

- Line 71: Probiotics activity is strain-specific. In the study referenced 11, the list of strains is not shown; tehrefore, there are fermenting bacteria, not probiotics.

We accepted this reviewer’s suggestion. We have inserted the word “fermented bacterial products” in the previous sentence (line 81).

- Line 95: What do authors mean: "improving GI environment", we do not know the healthy microbiota and only speculate about it.

We accepted this reviewer’s suggestion. Certainly no one knows a healthy microbiota. Therefore, it was rewritten to affect the GI environment (line 108).

- Line 109: With the used study design, it is impossible to distinguish whether the microbiota changes depended on LEX or training; this issue must be clearly stated in the discussion.

We accepted this reviewer’s suggestion. We have described the discussion section (line 432–434). It also clearly stated that a randomized, placebo-controlled trial should be conducted (line 440–443).

- Line 120: The composition of the product, the names of the strains and their quantities were not given.

Since the test product is a commercial product and does not contain live bacteria, we did not mention the name of the strains, but we added the number of bacteria at the time of fermentation, the extraction process, and the expected bacterial equivalent of the extract, according to the opinion of reviewer’s suggestion (line 137–140).

- Line 144: Why was urine not tested at the same time points as a stool?

It is necessary to refrain from consuming sports drinks and fruits that affect urine metabolites from the day before the urine collection. Therefore, considering the maintenance of the condition before the race, the urine collection on the day before the race could not be carried out at the same time as the fecal collection. We have described the discussion section (line 510–514).

- Results: How the authors related the compositionality problem?

Thank you for pointing out the viewpoint. We think that the gut bacterial composition (including its metabolites) and dietary intake are closely related. This study did not investigate accurate dietary intake (especially before and after the race). Fat and energy have been reported to be the largest connected components in the network between bacterial taxa. Changes in the bacterial composition may be induced to regulate energy and hormonal balance, or to respond to changes in the diet ingested. It is also thought that the mental stress of competition is also involved. In any case, the changes in the bacterial composition due to a single competition may have major impact on maintaining the athlete's post-competition condition. These perspectives have been added to the discussion section (line 480–493).

2. Reviewer 2

- 1. Abstract

“When the fecal microbiota was investigated before and after a race without the use of a supplement (pre-observation period), there was an increase in the abundance of phylum Bacteroidetes and decrease in the abundance of Firmicutes.” The opposite is mentioned in the results section as well as in Figure 2B.

We appreciate the reviewer’s comment. We collected the text (line 36 and 37).

■　2. Methods

a. General – statistical methods are poorly described – some of them, like exclusion of samples by Smirnov-Grubbs test (line 591) are only mentioned in results and/or figure description.

We appreciate the reviewer’s comment. We have added the description of statistical analysis (line 219–221). Also, since there was a lack of description of statistical methods for the item of exercise load, we added it (line 232–236).

b. Line 178 – software versions for usearch and QIIME should be given

We appreciate the reviewer’s comment. We have described the software versions for usearch and QIIME (line 194 and 196).

c. Line 181 – which OTU picking strategy from QIIME was used? What database was used to assign taxonomic classification?

We appreciate the reviewer’s comment. We used Greengenes 13_8 database, so, we have added it to the text (line 199).

d. Line 201 – no post-hoc test is identified for Friedman’s test

e. Line 202 – no mention of multiple comparison adjustment of p-value is given here yet it is present in the results

We appreciate the reviewer’s comment. We have described post-hoc test for Friedman’s test. We used a Scheffé's method　for Friedman’s test, as post-hoc test (line 224).

f. Line 204 –Friedman’s test (a non-parametric method) was used previously for differential analysis of microbial taxa which indicates the authors believe the taxa abundance distribution deviates from normal distribution. But here, Pearson’s correlation coefficient is computed which is best suited for variables with normal distribution. Could authors justify their choice? Wouldn’t Spearman correlation coefficient be better?

We appreciate the reviewer’s comment for statistical processing. Appropriate methods were not applied for some items for differential analysis of microbial taxa. The taxa abundance distribution of the phylum *Bacteroidetes* and *Firmicutes* were normal distribution. Therefore, we have changed to one-way ANOVA with Bonferroni’s correction (line 221–223, line 296–298). As for the F / B ratio, the normality was not recognized, so the Friedman ’s test was used (line 223–224, line298–299). Similarly, PCoA analysis confirmed normality, so we have changed to one-way ANOVA. Because the bacterial distribution was normal, the analysis of the relationship between urinary metabolites and bacterial composition remained Pearson’s correlation coefficient. Bacterial taxa and diversity analysis of variance in S2 Fig and S3 Fig were also normal, so we changed to ANOVA (line 651 and 659). We have changed some statistical analysis methods, but the results are not affected. Due to these changes, Fig 2(A and B), S2 Fig and S3 Fig have been replaced.

g. Line 205 and 201 – two different p-value adjustment strategies are used: FDR and Bonferroni. Could authors justify their choice here?

We appreciate the reviewer’s comment. If the number of multiple comparisons in the test was five or less, Bonferroni’s correction was used, and more than that, FDR was used. So, FDR was used to analyze the urinary metabolites and the relationship of bacterial composition and urinary metabolites, which have many items to be compared. Since the description was halfway, we have added it to the text (line 229–231).

- M&M: following the reagents, the information of company, city, country should be included.

It describes the reagents we used. Also, regarding the contract examination, the company is described.

■　3. Results

a. It would be good to see some baseline characteristics of athletes other than age, such as their BMI. Information on diet is also missing – even general remarks (was anyone on vegan/vegetarian diet? Was any other supplementation received?) would be helpful if such information is available.

We appreciate the reviewer’s comment. We added a paragraph describing the background of the participants (line 247–253).

- Minor remarks:

Some wording choice is unusual, such as Bonferroni’s correction coefficient (line 260). It would be better to say simply Bonferroni’s correction of Bonferroni’s p-value adjustement.

We accepted this reviewer’s suggestion (line 297).

3. Additional changes
(i) Changed the notation of Co-author affiliation. The affiliation at the time of the study and the current affiliation were separated.
(ii) We have an English proofreader correct the detailed English wording and change it. See revised manuscript with tracked changes.

We hope that we have addressed all the issues raised, and would be happy to clarify further on any other issues. Thank you for consideration of our manuscript for publication in your journal.

Yours sincerely,

Yasuhiro Sasuga
