# Supplementary material for: The impact of a competitive event and the efficacy of a lactic acid bacteria-fermented soymilk extract on the gut microbiota and urinary metabolites of endurance athletes: An open-label pilot study
Source: PLoS One. 2022 Jan 27;17(1):e0262906. doi: 10.1371/journal.pone.0262906 (PMC8794134; doi:10.1371/journal.pone.0262906)
Supplement: Attachment — Submitted filename: Response to Reviewers_R1.doc [file pone.0262906.s007.doc]

Krzysztof Durkalec-Michalski, Ph.D

Academic Editor

PLOS ONE

Yasuhiro Sasuga, Ph.D

Hachioji Center for Research & Development

B&S Corporation Co., Ltd.

17^th^ November 2021

Dear Dr. Durkalec-Michalski

**Response to reviewers’ comments [PONE-D-21-11683R1]**

Thank you for reviewing our manuscript and for the helpful comments provided.

Please find enclosed our response to the comments raised. The line numbers provided here are based on our revised manuscript with tracked changes.

1. Additional Editor Comments

1.	Line 27 – correct “resent” to “recent”.

We appreciate the editor’s comment. We collected the text (line 21).

2.	Line 66 – try to reduce even one "and" in this sentence - is repeated too often.

We appreciate the editor’s comment. We collected the text (line 56-57).

3.	Line 133 - The heading "TEST ARTICLE" is unclear. Consider Improving - Maybe "Supplementation". Likewise, in the later sections (e.g. lines 147-148) - the "test article" is misleading.

We appreciate the editor’s comment. We collected the text from article to supplement (line 119, 120, and 138).

4.	Lines 151-153 - Please add the necessary clarifications what exactly was and how "An official race" was conducted.

We appreciate the editor’s comment. We inserted the text (line 142-147).

5.	Line 164 – unclear part – authors wrote “and exercise intensity in order to evaluate exercise intensity” – do the authors mean “and exercise specificity in order to evaluate exercise intensity”? Please revise it.

We thank the editor’s comment. We collected the text from “intensity” to “specificity” (line 158).

6.	Lines 166-167 – unclear part – authors wrote “ exercise load of the race when participating in the race”. Maybe some of the terms "race" can be replaced with "exercise tests" or "competition".

We appreciate the editor’s comment. We collected the text from “race” to “competition” (line 160).

7.	The statements in the methodical part of biological material storage "as soon as possible" (lines 159 and 175) and are quite unstable and unclear - what does that mean? What was the criterion or the range of time?

We appreciate the editor’s comment. We collected to “immediately” (line 168, line201). We also added the storage condition (line 168-169, Line 201-202).

8.	Lines 216-241 - In the description of the statistical parts it should also be clearly indicated whether the minimum sample size has been achieved, bearing in mind some losses.

This study was a pilot trial with a small number of participants. Since some data could not be obtained at some points, we described how to handle them (Line 211-213).

9.	Line 354 - put "concentration" behind "arabinose".

We have already described 17.00 ± 5.21 (SD) mmol / mol Cr as the arabinose concentration after ingestion of LEX (line 367).

10.	Line 421 - the authors write "an official competition" - but what was the detailed specificity of these efforts. Were they real competitions in which competitors competed with other competitors (not participating in the project) or simulated competitions in which efforts imitating competitions were carried out?

It was the intention of real competitions. However, there is already research on athletic competition and gut microbiota, and this study was characterized by tracking changes in athletes' microbiota and urinary metabolites, so we changed the description to that (Line 441-443).

11.	Line 477 - try to use a more formal language and replace the word "fascinating".

We appreciate the editor’s comment. We collected to “interesting” (line 490).

12.	Lines 480-481 - the authors wrote "During long-distance running, the total energy of the body is very consumed" - this sentence is confusing. Do the authors mean that the body's energy expenditure is significant / elevated?

We appreciate the editor’s comment. We collected to describe that long-distance runner need sustained energy (Line 493-495).

13.	Line 484 - You should probably put "intake" behind “Fat and energy”

We appreciate the editor’s comment. We put “intake” (Line 498).

14.	Line 534 – change “is about 260km” to past tense.

We appreciate the editor’s comment. We collected (line 546).

15.	Lines 570-571 - the sentence, however, may be too optimistic despite the assurance that this is a hypothetical opinion of the authors. One should approach this even more carefully and point to "possible" or "potential" activation and that LEX ingestion "may be" mediated.

We appreciate the editor’s comment. We collected to “may be” (Line 580).

2. Reviewer 1

- Most of the comments have been taken into account by the authors. However, I maintain my opinion that the article should include a list of the strains included in the product tested. As shown for example in the work of van Baarlen P et al. (van Baarlen P et al. Differential NF-kappaB pathways induction by Lactobacillus plantarum in the duodenum of healthy humans correlating with immune tolerance. Proc Natl Acad Sci U S A. 2009;106(7):2371-6.) killed strains have their specific physiological activities and it can be suspected that it is strain dependent.

We appreciate the reviewer’s comment. We have described all strains (Line 123-125). Also, the description has been changed to novel genera to reflect the reclassification of the genus *Lactobacillus* (Zheng J et al. 2020).

（Reference）

・Zheng J, et al..: A taxonomic note on the genus Lactobacillus: Description of 23 novel genera, emended description of the genus Lactobacillus Beijerinck 1901, and union of Lactobacillaceae and Leuconostocaceae. Int J Syst Evol Microbiol. 2020 Apr; 70(4): 2782–2858.

- Authors should clarify the answer to the compositionality problem. Microbiome data are compositional (relative) and give no information about absolute abundances, regardless of normalization procedures (Gloor et al., 2017). Therefore relevant statistical methods, e.g., based on log-ratios must be used, to avoid false-positive results (Knight et al., 2018; Mandal et al.,2015; Weiss et al., 2017). Information on the increase in abundance of a particular bacterial genus must be linked to the identification of the reference point (i.e. another genus) against which this occurred (Christensen et al., 2009;Morton et al., 2019, 2017). (Christensen et al., 2009;Morton et al., 2019, 2017).

We accepted this reviewer’s suggestion. We conducted linear discriminant analysis (LDA) Effect Size (LEfSe) method (Segata N et al. 2011) to detect the differences in bacterial taxa characterizing the groups. There was no change in the basic data interpretation due to the change in the analysis method.

Due to the change of analysis method, the description of the method (Statistical analysis section)　has been changed (Line 214-216).

Due to the change of analysis method, figures have also changed (Fig 2B, add Fig 2C, S5 Fig). Figure legends have also been changed (Line 292-296, Line 671-682).

The result of S2 Fig was partially inappropriate as a result of LEfSe analysis, so it was replaced with the F / B ratio (S2 Fig., and Line 648-655).

In the result section, the description has been changed based on the result of LEfSe analysis (Line 308-331, Line 422-436).

The analysis of *Parabacteroides distasonis*, since LEfSe analysis was widely performed, exclusion by Smirnov-Grubbs test was not performed, and analysis was performed at n = 11.　 Therefore, the description of Smirnov-Grubbs test was deleted (Line 220, Line 432-433 Line 681-682).

Due to the change of analysis method, one reference has been added (reference No. 18) (Line 781-784). Therefore, subsequent reference numbers have changed.

（Reference）

Segata N, Izard J, Waldron L, Gevers D, Miropolsky L, Garrett WS, et al. Metagenomic biomarker discovery and explanation. Genome Biol. 2011;12(6):R60.

3. Additional changes

Organized the description in the Statistical analysis section.

◆Moved the software description to the end　（Line　240-242）.

◆Since ANOVA was a repeated measure, the description was changed from “one way ANOVA” to “repeated measures ANOVA” (Line 222, 302, 660).

◆The description of the F-test was missing, so we added it (Line 226-227).

Line 26: 16s → 16S

Line 195, 284: Unifrac → UniFrac

Line 405: tricarballyic → tricarballylic

Line 414: kynureic → kynurenic

Line 416: Added phylum and family of *Parabacteroides distasonis.*

Line 483: *Actinovateria* → *Actinobacteria*

Line 532: Additionaly → Additionally

Fig 1: Since the description of the number of analyzes in S5 FIg was missing, we added it. As a result, there are two “Fecal microbiota” in Data analysis, so we distinguished them (all and LEX).

We hope that we have addressed all the issues raised, and would be happy to clarify further on any other issues. Thank you for consideration of our manuscript for publication in your journal.

Yours sincerely,

Yasuhiro Sasuga
