# Supplementary material for: The impact of a competitive event and the efficacy of a lactic acid bacteria-fermented soymilk extract on the gut microbiota and urinary metabolites of endurance athletes: An open-label pilot study
Source: PLoS One. 2022 Jan 27;17(1):e0262906. doi: 10.1371/journal.pone.0262906 (PMC8794134; doi:10.1371/journal.pone.0262906)
Supplement: Attachment — Submitted filename: Response to Reviewers_R2.doc [file pone.0262906.s008.doc]

Krzysztof Durkalec-Michalski, Ph.D

Academic Editor

PLOS ONE

Yasuhiro Sasuga, Ph.D

Hachioji Center for Research & Development

B&S Corporation Co., Ltd.

10^th^ December 2021

Dear Dr. Durkalec-Michalski

**Response to reviewers’ comments [PONE-D-21-11683R2]**

Thank you for reviewing our manuscript and for the helpful comments provided.

Please find enclosed our response to the comments raised. The line numbers provided here are based on our revised manuscript with tracked changes.

1. Reviewer 3

- Minor revisions:
- 1- Line 218: Specify that the distribution was non-normal rather than “If failed.”

We appreciate the reviewer’s comment. We corrected to “non-normally distribution” (Line 218-219).

- 2- Line 219: Clarify that Scheffé refers to Scheffé’s multiple comparison method.

We appreciate the reviewer’s comment. We corrected to “Scheffé’s multiple comparison method” (Lines 219, 630-631)

- 3- Line 221: Pearson’s correlation coefficients are used to illustrate the linear relationship between two variables. Additionally, graphs are provided. Pearson’s correlation coefficients are not obvious by inspecting graphs. Rephrase this sentence to improve clarity.

We appreciate the reviewer’s comment. We rephrased this sentence (Lines 221-223).

- 4- Lines 224-226: Clarify the use of the Wilcoxon signed-rank test. The term “non-parametric” can be dropped as a descriptor for Wilcoxon signed-rank and Freidman’s tests since no parametric versions of these tests exist.

We appreciate the reviewer’s comment. We dropped “non-parametric” (Lines 219, 228).

- 5- Lines 227-230: Drop the portion of the sentence following the colon.

We appreciate the reviewer’s comment. We dropped the portion of the sentence following the colon (Lines 231-233).

- 6- Line 240: Replace “background” with “baseline characteristics.”

We appreciate the reviewer’s comment. We replaced “background” with “baseline characteristics.” (Line 242).

- 7- Line 241-243: Upfront, state that the mean and standard deviation are being summarized. No need to repeat SD each time.

We appreciate the reviewer’s comment. We corrected (Lines 243-245).

- 8- Line 245: Provide the percentage that corresponds to 8 of 13.

We appreciate the reviewer’s comment. We described the percentage　（Line 248）.

- 9- Lines 254-257: Clarify this sentence.

We appreciate the reviewer’s comment.　We rephrased this sentence to improve clarity (Lines 257-259).

- 10- Line 266: Are the results statistically significant? If so, support the statement with a p-value.

We appreciate the reviewer’s comment.　We described p-value (Lines 271-273).

- 11- The standard statistical term for average is mean.

We appreciate the reviewer’s comment. We replaced “average” with “mean.” (Lines 278, 298, 508, 525, 622, 629, 635, 652).

- 12- Lines 274 -280: Clarify PRE_a, PRE_b, POST_a, POST_b.

We appreciate the reviewer’s comment. It has described in materials & methods. However, we added explanations to improve clarity (Lines 280-281).

- 13- Provide p-values to support statements indicating statistical significance.

We appreciate the reviewer’s comment.　We described p-value (Lines 310, 320, 352, 360-361, 363-366).

- 14- Indicate the date range subjects were enrolled in the study.

We appreciate the reviewer’s comment. We described the date range subjects were enrolled (Line 135).

- 15- The p-value associated with a correlation is a test of the null hypothesis: correlation equal to zero; however, the absolute magnitude of the coefficient indicates the strength of the linear relationship between two variables. In general, the strength or correlation coefficient is the more important statistic to focus on.
- Below is a table for interpreting correlation coefficients:
- Coefficient (absolute value) Interpretation
- 0.90 - 1.0 Very Strong
- 0.70 - 0.89 Strong
- 0.40 - 0.69 Moderate
- 0.10 - 0.39 Weak
- less than 0.10 Negligible correlation

We appreciate the reviewer’s comment. Focusing on the correlation coefficient, we described the coefficient interpretation (Lines 38, 376, 399, 405).

We dropped “significant” (Lines 378, 383, 388).

We added coefficient (absolute value) (Lines 401-403, 406-409).

2. Additional changes

Organized the description in the Statistical analysis section.

Line 225: relationship → associations

Line 279, 284: delete “SD”

Line 348: delete “average”

Line 352: delete “SD”

Lines 407-408: delete “phenylalanine and tyrosine metabolites”*.*

Lines 408-409: delete “tryptophan metabolites”*.*

Line 409: delete “uracil”

Line 645: 9 → nine

Multi-panel Figures: place all panels from a multi-part figure into a single page and single file (Fig 1, Fig 2).

We hope that we have addressed all the issues raised, and would be happy to clarify further on any other issues. Thank you for consideration of our manuscript for publication in your journal.

Yours sincerely,

Yasuhiro Sasuga
